# Beyond the Pocket: Clinical, Routine Biomarker, and Microbiological Associations with Systemic Versus Localized Cardiovascular Implantable Electronic Device Infection

**DOI:** 10.3390/diagnostics16183062

**Published:** 2026-09-21

**Authors:** Dilek Karamanlıoğlu, Murat Akdoğan, Özcan Özdemir, Hamza Sunman, Tolga Han Efe, Engin Algül, Çağatay Tunca, Gülnur Kul, Yunus Gürbüz, Murat Karamanlıoğlu

**Affiliations:** 1 Department of Infectious Diseases and Clinical Microbiology, Etlik City Hospital, 06170 Ankara, Türkiye; gkul2004@gmail.com (G.K.); yunusgurbuz@outlook.com (Y.G.); 2 Department of Cardiology, Etlik City Hospital, 06170 Ankara, Türkiye; drmuratakdogann@gmail.com (M.A.); drozdemir75@yahoo.com (Ö.Ö.); hamzasunman@gmail.com (H.S.); medisay@gmail.com (T.H.E.); algulengin@gmail.com (E.A.); md.tunca@gmail.com (Ç.T.); 3 Department of Cardiology, Ankara Atatürk Sanatorium Training and Research Hospital, University of Health Sciences, 06280 Ankara, Türkiye; drmurat.krm@gmail.com

**Keywords:** cardiac implantable electronic device infection, pocket infection, systemic infection, procalcitonin, fever, device extraction, microbiology, mortality

## Abstract

**Background/Objectives**: Cardiac implantable electronic device (CIED) infections range from localized pocket infection to systemic disease. This study evaluated clinical, laboratory, microbiological, and imaging findings associated with the final classification of systemic CIED infection and described device management and in-hospital outcomes. **Methods:** This retrospective single-center study included 75 adults with definite CIED infection after case-by-case diagnostic re-adjudication, comprising 61 patients with localized pocket infection and 14 with systemic infection. Exploratory univariable and Firth penalized logistic regression analyses were used to examine associations with systemic infection. **Results:** Of 75 patients with definite CIED infection, 14 (18.7%) had systemic infection and 61 (81.3%) had localized pocket infection. Fever was observed in 5/14 (35.7%) patients with systemic infection and 3/61 (4.9%) patients with localized infection (*p* = 0.005). Procalcitonin was available for 70 patients and was higher in systemic than in localized infection (0.23 [0.06–1.57] vs. 0.05 [0.03–0.12] ng/mL; *p* = 0.025). Chronic kidney disease stage G3a–G5 was more frequent in systemic infection (3/14 [21.4%] vs. 2/61 [3.3%]; *p* = 0.042), and estimated glomerular filtration rate was lower (64 [45–83] vs. 89 [73–99] mL/min/1.73 m^2^; *p* = 0.014). Among 33 culture-positive episodes, coagulase-negative staphylococci were recovered in 12/22 localized episodes, whereas *Staphylococcus* *aureus* was recovered in 6/11 systemic episodes. Complete system removal was documented in 12/14 (85.7%) systemic and 46/61 (75.4%) localized infections; the median interval from admission to first removal attempt was 10 [6–17] and 5 [3–8] days, respectively. In the exploratory Firth model including age, fever, and log_2_-transformed procalcitonin, fever (aOR 6.17, 95% CI 1.02–37.52) and procalcitonin per doubling (aOR 1.35, 95% CI 1.02–1.81) were associated with systemic infection. In the kidney-function sensitivity model, the procalcitonin estimate was attenuated (aOR 1.31, 95% CI 0.99–1.74). Two in-hospital deaths occurred, both among patients with systemic infection. **Conclusions:** In this small retrospective cohort, fever and higher procalcitonin concentrations were exploratory correlates of the systemic-infection classification. The study did not evaluate diagnostic accuracy, clinical prediction, calibration, or external validity. Larger prospective studies with standardized diagnostic work-up and external validation are needed before biomarker-based clinical decisions can be considered.

## 1. Introduction

Cardiovascular implantable electronic devices (CIEDs), including permanent pacemakers, implantable cardioverter-defibrillators, and cardiac resynchronization therapy systems, are widely used in the management of bradyarrhythmia, prevention of sudden cardiac death, and treatment of selected patients with heart failure. The expanding indications for these devices and increasing life expectancy have resulted in a growing population living with CIEDs. However, device-related infection remains one of the most serious complications because it is associated with prolonged hospitalization, complex treatment, substantial healthcare costs, and increased mortality [1,2]. Although CIED infection develops after approximately 1% of procedures, its occurrence reflects the interaction of patient-related, procedure-related, and device-related predisposing conditions [2,3]. Patient-related factors associated with a higher overall risk of CIED infection include diabetes mellitus, chronic kidney disease, heart failure, malignancy, corticosteroid or other immunosuppressive treatment, and antithrombotic therapy [4,5,6]. At the procedure and device level, repeated pocket manipulation, generator replacement, lead revision, system upgrade, temporary pacing, prolonged procedure duration, inadequate peri-procedural antimicrobial prophylaxis, and postoperative pocket hematoma have been associated with increased infection risk [4,5,6,7].

CIED infections encompass a broad clinical spectrum ranging from superficial incisional infection to isolated generator-pocket infection, lead infection, bloodstream infection, and CIED-related infective endocarditis. Pocket infection may present with erythema, pain, swelling, fluctuance, purulent drainage, wound dehiscence, sinus tract formation, or device erosion. Systemic infection may occur with or without pocket findings and can manifest as persistent bacteremia, fever of unknown origin, sepsis, lead vegetation, tricuspid valve involvement, or septic pulmonary embolism [1,8]. The absence of visible pocket abnormalities may delay the diagnosis, particularly in older, immunocompromised, or previously treated patients.

CIED infections develop primarily through contamination of the generator pocket or leads by skin flora during implantation or a subsequent device-related procedure [6]. Less frequently, an initially sterile device may become infected through hematogenous seeding during bacteremia originating from a distant focus [9]. After adhering to the device surface, microorganisms can form biofilms that reduce their susceptibility to host immune responses and antimicrobial agents. Biofilm formation promotes persistent infection and explains why antibiotic therapy without removal of infected hardware is frequently unsuccessful [4,10,11].

Gram-positive bacteria, particularly staphylococci, cause most microbiologically confirmed CIED infections. Coagulase-negative staphylococci are commonly associated with indolent pocket infections, whereas *Staphylococcus aureus* is more frequently associated with bacteremia, infective endocarditis, and adverse outcomes. Other causative microorganisms include *Enterococcus* species, streptococci, *Cutibacterium acnes*, *Corynebacterium* species, and, less commonly, Gram-negative bacilli and fungi [12]. A multicenter study from Türkiye similarly identified generator-pocket infection as the most common clinical presentation and coagulase-negative staphylococci and *S. aureus* as the leading causative pathogens [13]. Nevertheless, the distribution of microorganisms and their antimicrobial susceptibility patterns may differ across institutions and geographical regions.

The development and clinical course of CIED infection are influenced by patient-, procedure-, and device-related factors. Diabetes mellitus, chronic kidney disease, end-stage renal disease, chronic obstructive pulmonary disease, heart failure, malignancy, corticosteroid use, anticoagulant therapy, previous device infection, and preprocedural fever have been associated with increased infection risk [14,15,16,17]. Important procedure-related factors include the absence of antimicrobial prophylaxis, prolonged procedure duration, temporary pacing, early reintervention, generator replacement, system revision or upgrade, and postoperative pocket hematoma [1,2,7,16,18,19,20]. Pocket hematoma is particularly important because it is potentially preventable and is strongly associated with subsequent device infection [7]. Careful patient selection, preoperative antimicrobial prophylaxis, strict aseptic technique, appropriate management of antithrombotic therapy, and prevention of hematoma are therefore essential components of infection prevention.

The standard treatment for definite pocket infection, lead infection, or CIED-related infective endocarditis consists of complete removal of the generator and all leads combined with appropriate systemic antimicrobial therapy [1,21,22]. Conservative approaches involving antimicrobial therapy alone, limited pocket debridement, or generator relocation leave infected foreign material in place and are associated with persistent or recurrent infection. Early device removal is also clinically important. More recent nationwide data demonstrated that delayed lead removal was associated with increased in-hospital mortality, major adverse events, and longer hospitalization [23].

Microbiological characterization has direct therapeutic relevance in CIED infection because empirical antimicrobial treatment should provide appropriate coverage for the most likely pathogens and subsequently be refined according to culture and susceptibility results. In patients with definite CIED infection, pathogen-directed systemic antimicrobial therapy should be combined with complete device and lead removal whenever feasible, because antimicrobial treatment alone may fail when infected hardware is retained [12,21,22]. Published cohorts have also shown that systemic involvement and delays in definitive source control are associated with less favorable clinical outcomes, including prolonged hospitalization and increased mortality [21,23]. Accordingly, characterization of microbiological findings, device management, and short-term outcomes remains clinically important in real-world CIED infection cohorts.

Despite advances in prevention, diagnosis, and extraction techniques, contemporary data regarding the clinical presentation, microbiological findings, device management, and short-term outcomes of CIED infection in Türkiye remain limited. Therefore, this study aimed to compare the clinical presentation, routine inflammatory biomarkers, microbiological findings, echocardiographic evaluation, device management, and in-hospital outcomes of localized pocket infection and systemic CIED infection, and to explore pre-classification factors associated with systemic involvement.

## 2. Materials and Methods

### 2.1. Study Design and Setting

This retrospective, single-center observational study used routinely collected clinical records from patients treated between September 2022 and September 2025. No study-specific intervention or prospective research procedure was undertaken during the clinical observation period. The protocol for the retrospective analysis of de-identified data was approved by the Ankara Etlik City Hospital Scientific Research Evaluation and Ethics Committee on 17 December 2025 (Decision No. AEŞH-BADEK1-2025-763). Written informed consent was obtained from all participants included in the study. All data were anonymized before analysis, and the study was conducted in accordance with the principles of the Declaration of Helsinki.

Patients hospitalized in the cardiology ward or intensive care units were identified through the hospital electronic medical record system. The study was reported in accordance with the Strengthening the Reporting of Observational Studies in Epidemiology (STROBE) recommendations.

Patients were eligible if they were aged ≥ 18 years, had a CIED in situ, and were diagnosed with generator-pocket infection, lead infection, or CIED-related infective endocarditis. CIED infection was evaluated according to the European Heart Rhythm Association consensus criteria and the 2023 European Society of Cardiology guidelines [2,8]. Patients with isolated superficial incisional inflammation without pocket or hardware involvement were not classified as having CIED infection. Records lacking sufficient clinical, microbiological, imaging, or outcome data to confirm the diagnosis were excluded.

All eligible patients identified during the study period were included, resulting in a preliminary analytic cohort of 82 patients. Because the study used all available cases within the predefined period, no formal a priori sample-size calculation was performed.

### 2.2. Definitions and Classification of Infection

Pocket infection was defined by the presence of local inflammatory findings involving the generator pocket, including erythema, swelling, tenderness, discharge, wound dehiscence, fluctuance, sinus tract formation, or device erosion [1,2,8]. Ultrasonographic evidence of a pocket collection or hematoma was recorded when superficial ultrasonography had been performed.

Systemic CIED infection was defined as device-attributable microbiologically confirmed bloodstream infection and/or echocardiographic evidence of lead or valvular vegetation, in accordance with contemporary AHA, EHRA, and ESC guidance [1,2,8]. CIED-related infective endocarditis was diagnosed using compatible clinical, microbiological, and imaging findings according to the 2023 ESC criteria [8]. Patients with local pocket findings without bacteremia, lead vegetation, or valvular involvement were classified as having localized pocket infection.

Culture-positive infection was defined as the isolation of a clinically relevant microorganism from wound or pocket discharge, intraoperative pocket tissue or extracted lead material, or peripheral blood cultures. This prespecified operational definition was consistent with contemporary CIED-infection guidance regarding microbiological confirmation [1,2]. Cases in which adequate microbiological samples yielded no growth were classified as culture-negative infections.

All cases initially retained in the analytic cohort underwent case-by-case diagnostic re-adjudication using prespecified criteria derived from the EHRA International CIED Infection Criteria and the 2023 ESC Guidelines [2,8]. Classification was based on the index clinical presentation, evidence of generator-pocket or hardware involvement, microbiological findings, imaging, operative records when available, and assessment of alternative infection sources; device removal status and timing from implantation were not used as diagnostic criteria.

Superficial incisional infection was defined as acute inflammation confined to the skin and subcutaneous tissue without involvement of the generator pocket or CIED hardware and was excluded. Definite localized pocket infection required documented evidence of pocket or hardware involvement, such as purulent drainage, sinus formation, pocket deformation or adherence, threatened erosion, device or lead exposure, a documented pocket collection, or compatible intraoperative findings, without evidence of systemic involvement. Cases in which the available documentation did not permit reliable differentiation between superficial inflammation and definite CIED infection were classified as possible or indeterminate and were excluded from the primary analytic cohort.

### 2.3. Data Collection

Demographic and clinical data were retrospectively extracted using a standardized data-collection form. The recorded variables included age, sex, and the interval between the most recent device procedure and presentation with infection. Local and systemic clinical findings included fever, pocket discharge, edema, tenderness, erythema, wound dehiscence, ultrasonographically detected collection, and hematoma.

Recorded comorbidities included diabetes mellitus, hypertension, chronic kidney disease, chronic obstructive pulmonary disease, human immunodeficiency virus infection, coronary artery disease, and heart failure. Additional comorbid conditions documented in the medical records were also recorded. Heart failure was defined as a clinician-documented diagnosis supported by the medical record and available echocardiographic findings. Where an interpretable baseline left ventricular ejection fraction (LVEF) was available, heart failure was categorized as heart failure with reduced ejection fraction (HFrEF; LVEF ≤ 40%), mildly reduced ejection fraction (HFmrEF; LVEF 41–49%), or preserved ejection fraction (HFpEF; LVEF ≥ 50% with documented clinical heart failure and compatible structural heart disease, diastolic dysfunction, or elevated filling-pressure findings) [24]. The most recent pre-index or clinically stable echocardiographic assessment was used whenever available. New York Heart Association (NYHA) functional class was obtained from the most recent stable clinical assessment before the index infection episode. Acute decompensated heart failure was recorded when new or worsening congestion or dyspnea required intravenous diuretic, vasodilator, or inotropic treatment, or hospitalization for heart-failure management, during the index presentation. For patients with cardiac resynchronization therapy (CRT), device type and the documented original indication for CRT were recorded.

Chronic kidney disease was defined by a documented chronic diagnosis with evidence of kidney abnormality persisting for at least 3 months, including an estimated glomerular filtration rate (eGFR) < 60 mL/min/1.73 m^2^, or by maintenance dialysis [25]. Baseline CKD stage was categorized according to the most recent stable pre-index eGFR as G3a (45–59), G3b (30–44), G4 (15–29), or G5 (<15 mL/min/1.73 m^2^); dialysis-dependent kidney disease was recorded separately. The eGFR measured at admission or initial infection assessment was analyzed descriptively but was not used alone to establish chronic kidney disease. Fever was defined as a body temperature ≥38.0 °C documented at admission or initial infection assessment before initiation of new infection-directed antimicrobial treatment, when this timing was available.

Laboratory variables obtained at the time of admission or initial infection assessment included leukocyte count, hemoglobin, platelet count, erythrocyte sedimentation rate, C-reactive protein, procalcitonin, and glycated hemoglobin. Laboratory measurements were analyzed using the first available value obtained before or at the initiation of infection-directed treatment. To facilitate clinical interpretation, each available laboratory result was additionally categorized as below, within, or above the contemporaneous adult institutional reference interval recorded for that test. Sex-specific reference intervals were applied for hemoglobin and, where applicable, age- and sex-specific reference intervals were applied for erythrocyte sedimentation rate. Assay-specific upper reference limits were used for C-reactive protein and procalcitonin.

### 2.4. Echocardiographic and Microbiological Evaluation

Transthoracic echocardiography findings were reviewed for all patients in whom the examination was performed. Transesophageal echocardiography was performed when clinically indicated, particularly in patients with bacteremia, suspected lead involvement, or inconclusive transthoracic findings. Echocardiographic reports were reviewed for the presence and anatomical location of vegetations, including lead, tricuspid, mitral, aortic, or vena cava involvement.

Available microbiological samples included cultures obtained from pocket discharge, wound material, intraoperative pocket tissue or extracted device components, and peripheral blood. Microorganisms were identified and antimicrobial susceptibility was evaluated according to the routine procedures and interpretive criteria used by the hospital microbiology laboratory during the study period. Isolates were categorized as methicillin-susceptible *Staphylococcus aureus*, methicillin-resistant *S. aureus*, methicillin-resistant coagulase-negative staphylococci, methicillin-susceptible coagulase-negative staphylococci, *Enterococcus* species, Gram-negative bacilli, fungi, or other microorganisms.

### 2.5. Device Management and Outcomes

Device-related management during the index hospitalization was re-abstracted from operative reports, extraction reports, and discharge summaries. Management was classified as: (i) complete system removal, defined as removal of the pulse generator and all transvenous leads without a documented retained lead fragment; (ii) incomplete or partial system removal, defined as a removal attempt with retained lead material or another device component; (iii) pocket debridement without system removal; or (iv) conservative treatment without an attempted system removal. The term “any system removal” includes both complete and incomplete or partial system removal.

For patients who underwent a removal attempt, time to first removal was calculated from the date of index hospital admission for the CIED infection episode to the date of the first removal attempt. A diagnosis-to-removal interval was not used. When available, the extraction approach (transvenous, surgical, or hybrid), documented procedure-related complications, reimplantation strategy, and the documented reason for non-extraction were recorded. Detailed antimicrobial regimen, route, timing, total duration including outpatient therapy, clinical or microbiological response, and the case-specific indication for individual removal procedures were not consistently available in a standardized form. Device-management findings were therefore interpreted descriptively and were not used as markers of antimicrobial treatment failure.

### 2.6. Statistical Analysis

All analyses were performed in the final re-adjudicated cohort of patients with definite CIED infection. Statistical analyses were conducted using IBM SPSS Statistics version 27.0 (IBM Corp., Armonk, NY, USA) and R software version 4.3.0 (R Foundation for Statistical Computing, Vienna, Austria). The distribution of continuous variables was assessed using the Shapiro–Wilk test, histograms, and Q–Q plots. Normally distributed continuous variables are presented as mean ± standard deviation, whereas non-normally distributed variables are presented as median [interquartile range]. Categorical variables are presented as number (%). Patients with localized pocket infection and systemic CIED infection were compared using the independent-samples *t*-test or Mann–Whitney U test for continuous variables, as appropriate, and the Pearson chi-square test or Fisher’s exact test for categorical variables. Analyses of microbiological variables contributing directly to the operational definition of systemic infection, including blood-culture positivity and the composite variable of any positive microbiological culture, were descriptive only and were not interpreted as independent between-group associations.

Associations with the final classification of systemic CIED infection were examined using univariable Firth penalized logistic regression. Because only 14 systemic-infection events were available, Firth penalization was used to reduce sparse-data bias and the number of covariates in multivariable models was deliberately restricted to reduce overfitting [12]. Candidate variables were selected a priori on the basis of clinical relevance, data completeness, and the number of available events; automated stepwise selection was not used. Odds ratios (ORs) and adjusted odds ratios (aORs) are reported with 95% confidence intervals (CIs). Procalcitonin and time since the most recent CIED procedure were log_2_-transformed; their ORs therefore represent the change in odds associated with a twofold increase.

The primary exploratory multivariable model (Model A) included age, fever at presentation, and log_2_-transformed procalcitonin in the complete-case procalcitonin subset. An exploratory kidney-function sensitivity model (Model B) included chronic kidney disease stage G3a–G5, fever, and log_2_-transformed procalcitonin to assess potential confounding by kidney dysfunction. The available number of events did not permit simultaneous adjustment for additional potential confounders, including heart-failure severity, immune status, and other comorbidities. Blood-culture positivity, echocardiographic vegetation, transesophageal echocardiography use, microbiological isolate, device-management variables, and in-hospital mortality were not entered into regression models because they contributed to diagnostic classification, reflected clinician-directed diagnostic work-up, or occurred after the index clinical presentation.

To examine the potential influence of differential diagnostic work-up, a restricted sensitivity analysis was performed among patients with at least three peripheral blood-culture sets obtained before new antimicrobial treatment and both transthoracic and transesophageal echocardiography performed during the index episode. Findings in this restricted subgroup were summarized descriptively because the number of events was insufficient for multivariable modelling. Because only two in-hospital deaths occurred, mortality was summarized descriptively and no between-group mortality inference or multivariable mortality model was undertaken. Missing data were not imputed; analyses used available cases, and the applicable denominator is reported where necessary. All tests were two-sided. *p*-values are presented as exploratory measures of statistical compatibility with the null hypothesis; no adjustment for multiple comparisons was applied. The regression analyses were exploratory association analyses and were not intended to develop or validate a diagnostic or clinical prediction model. Accordingly, no area under the receiver-operating-characteristic curve, sensitivity, specificity, calibration, biomarker cut-off, or external-validation analysis was performed.

## 3. Results

Among 102 patients screened for suspected CIED infection, 20 were excluded at initial screening because of age < 18 years (n = 2), insufficient diagnostic documentation (n = 11), or superficial incisional infection without pocket or hardware involvement (n = 7). The preliminary analytic cohort comprised 82 patients, including 68 with localized pocket infection and 14 with systemic CIED infection.

**Figure 1:**
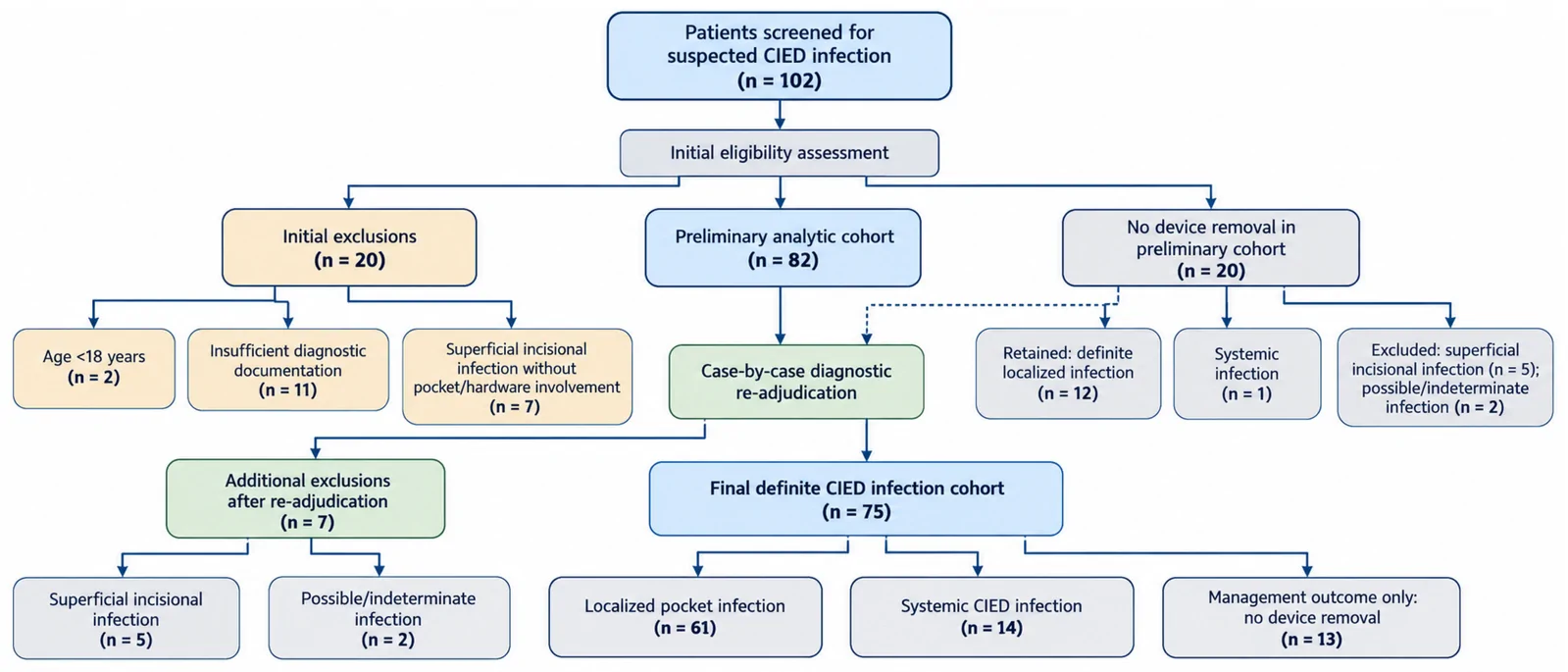
Flow diagram of patient screening, case-by-case diagnostic re-adjudication, and final classification of the cohort. Device removal was analyzed as a descriptive management outcome and was not used as a diagnostic criterion.

All 82 cases underwent case-by-case diagnostic re-adjudication using the prespecified definitions. Seven additional patients were excluded from the primary analytic cohort: five were reclassified as having superficial incisional infection without pocket or hardware involvement, and two were classified as having possible or indeterminate infection not meeting the criteria for definite CIED infection. Of the 20 patients without documented device removal in the preliminary cohort, 12 had definite localized pocket infection and 1 had systemic CIED infection and were retained, whereas 5 had superficial incisional infection and 2 had possible or indeterminate infection. The final definite CIED infection cohort comprised 75 patients, including 61 (81.3%) with localized pocket infection and 14 (18.7%) with systemic CIED infection (Figure 1). Detailed diagnostic re-adjudication of the 20 patients without documented device removal in the preliminary cohort is presented in Supplementary Table S1.

Following diagnostic re-adjudication, the final analytic cohort comprised 75 patients with definite CIED infection, including 61 patients with localized pocket infection and 14 with systemic CIED infection. Patients with systemic infection were numerically older than those with localized infection (71.6 ± 11.5 vs. 65.7 ± 13.4 years, *p* = 0.143), whereas sex distribution, device type, time since the most recent CIED procedure, diabetes mellitus, hypertension, and coronary artery disease did not differ significantly between groups. Heart failure was more frequent in the systemic-infection group (92.9% vs. 62.3%, *p* = 0.029), as were chronic kidney disease stage G3a–G5 (21.4% vs. 3.3%, *p* = 0.042); estimated glomerular filtration rate was lower in systemic infection (64 [45–83] vs. 89 [73–99] mL/min/1.73 m^2^, *p* = 0.014). Fever at presentation was observed in 5 of 14 patients (35.7%) with systemic infection and in 3 of 61 patients (4.9%) with localized pocket infection (*p* = 0.005). Procalcitonin was available for 70 patients and was higher in systemic than localized infection (0.23 [0.06–1.57] vs. 0.05 [0.03–0.12] ng/mL, *p* = 0.025). Pocket drainage was less frequent in systemic infection than in localized pocket infection, although the difference was not statistically significant (35.7% vs. 57.4%, *p* = 0.234) (Table 1).

Among the 51 patients with heart failure, HFrEF, HFmrEF, and HFpEF accounted for 37.3%, 21.6%, and 41.2% of cases, respectively. LVEF was available for 48 patients with heart failure and was numerically lower in the systemic-infection group than in the localized-infection group (40 [30–48]% vs. 48 [36–56]%). NYHA class III–IV and acute decompensated heart failure at the index presentation were documented in 6 of 12 and 3 of 13 evaluable systemic-infection cases, respectively. CRT devices were present in 15 patients; the recorded indication was chronic heart failure with electrical desynchrony in 13 patients and anticipated high right-ventricular pacing burden or another indication in 2 patients. Kidney-function phenotyping showed CKD G3a–G5 in five patients, including three patients with systemic infection. One systemic-infection patient had advanced CKD with dialysis dependence. Detailed heart-failure and kidney-disease characteristics are provided in Supplementary Table S2.

**Figure 2:**
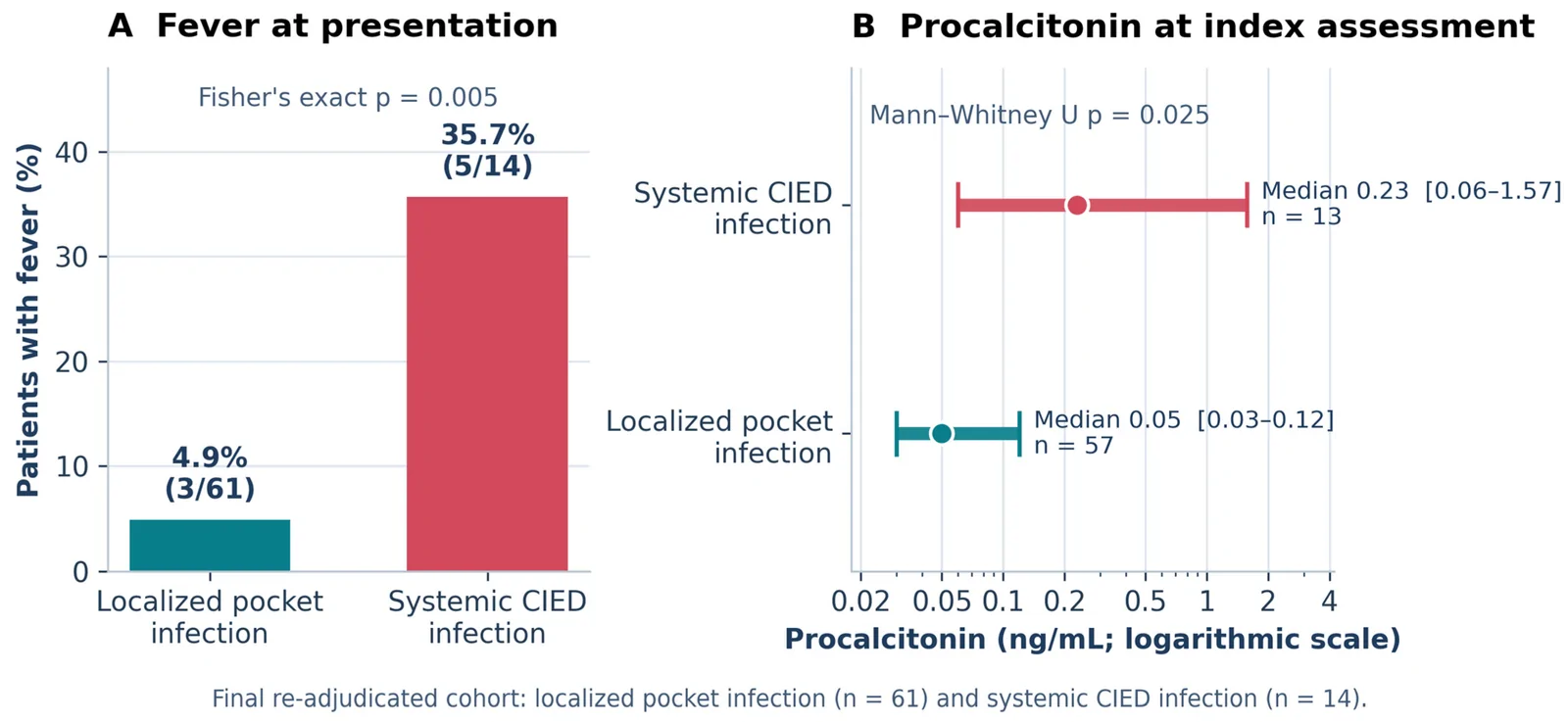
Fever at presentation and procalcitonin concentration according to final CIED infection classification.

Patients with systemic CIED infection presented with fever more frequently than those with localized infection (35.7% vs. 4.9%, *p* = 0.005). Procalcitonin concentrations were also higher in the systemic-infection group (median, 0.23 [0.06–1.57] vs. 0.05 [0.03–0.12] ng/mL; *p* = 0.025); procalcitonin was available in 13 of 14 systemic and 57 of 61 localized cases. Transesophageal echocardiography was performed more frequently in patients with systemic infection (85.7% vs. 21.3%, *p* < 0.001). The remaining clinical, laboratory, and imaging findings are summarized in Table 2. The distribution of inflammatory biomarkers according to final infection classification is presented in Figure 2.

**Figure 3:**
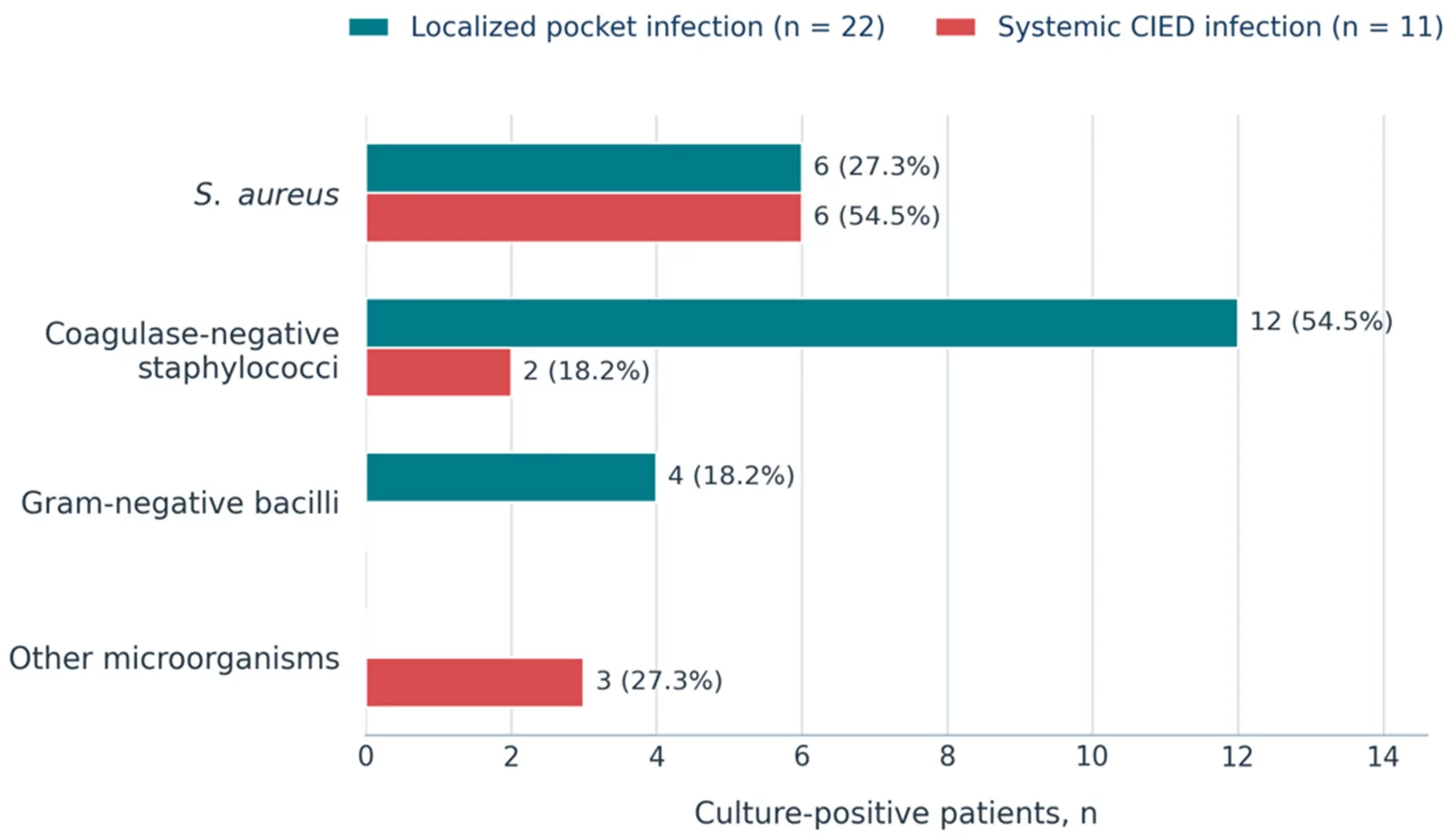
Distribution of microorganisms recovered from culture-positive CIED infection episodes according to final infection classification.

Microbiological findings are presented descriptively by specimen source in Table 3. Because positive blood-culture results contributed to the operational classification of systemic CIED infection, blood-culture positivity and the composite variable of any positive microbiological culture were not treated as independent between-group outcomes. Among the 33 culture-positive patients, coagulase-negative staphylococci predominated in localized pocket infection (12/22 culture-positive localized cases), whereas *Staphylococcus aureus* was the most frequently recovered organism in systemic infection (6/11 culture-positive systemic cases). The distribution of recovered microorganisms according to final infection classification is shown in Figure 3.

Detailed device-management findings are presented in Table 3. Any system removal was performed in 62 of 75 patients (82.7%), including 49 of 61 patients (80.3%) with localized pocket infection and 13 of 14 patients (92.9%) with systemic CIED infection. In the re-abstracted management classification, complete system removal was documented in 58 patients, whereas four patients underwent incomplete or partial removal with retained hardware. Pocket debridement without system removal was performed in three patients, and ten patients received conservative treatment without an attempted system removal. Among patients who underwent any system removal, the median time from index hospital admission to the first removal attempt was 6 [IQR, 3–9] days overall, 5 [IQR, 3–8] days in localized infection, and 10 [IQR, 6–17] days in systemic infection (*p* = 0.026). Because antimicrobial treatment details, clinical response, and case-specific reasons for management selection were incompletely available, these findings are descriptive and should not be interpreted as evidence that prior antimicrobial therapy was ineffective (Table 3).

In univariable Firth penalized logistic regression analyses, fever at presentation (OR 10.74, 95% CI 2.17–53.20; *p* = 0.003), chronic kidney disease stage G3a–G5 (OR 7.12, 95% CI 1.09–46.40; *p* = 0.042), and higher procalcitonin concentration per doubling (OR 1.46, 95% CI 1.11–1.94; *p* = 0.008) were associated with systemic CIED infection. Age, male sex, diabetes mellitus, heart failure, time since the most recent CIED procedure, and pocket drainage did not meet the prespecified threshold for statistical significance in univariable analyses. Complete procalcitonin data were available for 70 patients, including 13 patients with systemic CIED infection. In Model A, which included age, fever at presentation, and log_2_-transformed procalcitonin, fever (aOR 6.17, 95% CI 1.02–37.52; *p* = 0.048) and procalcitonin per doubling (aOR 1.35, 95% CI 1.02–1.81; *p* = 0.039) remained associated with systemic infection, whereas age was not statistically significant. In the exploratory kidney-function sensitivity model (Model B), the adjusted estimates were 4.15 (95% CI 0.39–44.00; *p* = 0.240) for chronic kidney disease, 5.82 (95% CI 0.91–37.00; *p* = 0.064) for fever, and 1.31 (95% CI 0.99–1.74; *p* = 0.058) for procalcitonin per doubling (Table 4).

Clinical presentation and routine hematological variables were complete for all patients, except for heart-failure status in one patient. Missingness was highest for HbA1c (50.0%) and erythrocyte sedimentation rate (41.5%), whereas procalcitonin was unavailable in five patients (6.1%). Imaging findings and microbiological results were nearly complete among patients who underwent the corresponding diagnostic procedures. Availability and missingness of clinical, laboratory, imaging, and microbiological variables are showed in Supplementary Table S3.

The categorical distribution of available laboratory results relative to institutional reference intervals is provided in Supplementary Table S4.

Diagnostic work-up and bloodstream-infection adjudication are summarized in Supplementary Table S5. Blood cultures were obtained in 58 of 75 patients, including 44 of 61 patients with localized pocket infection and all 14 patients with systemic CIED infection. At least three peripheral blood-culture sets obtained before new antimicrobial treatment were documented in 29 patients. TTE and TEE were performed in 73 and 25 patients, respectively; TEE was undertaken more frequently in patients classified with systemic infection (12/14) than in those with localized infection (13/61). As diagnostic testing was clinician-directed, tests not performed or not reliably reconstructable were coded as not assessed and were not interpreted as negative.

Eight patients had clinically significant positive blood cultures. Following case-by-case review of microbiological, imaging, operative, and alternative-source data, all eight episodes were classified as device-attributable bloodstream infection; six additional systemic cases were classified on the basis of compatible imaging and clinical evidence without positive blood cultures. Because positive blood cultures and echocardiographic vegetation contributed to the operational classification of systemic infection, blood-culture positivity and the composite variable of any positive microbiological culture were interpreted descriptively rather than as independent between-group outcomes.

The strict-verification sensitivity cohort comprised 20 patients who had at least three pre-antibiotic peripheral blood-culture sets and both TTE and TEE during the index episode, including 8 patients with localized pocket infection and 12 with systemic CIED infection. Within this restricted subgroup, fever remained more frequent and procalcitonin concentrations remained higher in systemic infection; however, the small number of strictly verified systemic cases precluded multivariable modelling. These sensitivity findings were therefore considered exploratory and do not eliminate residual bias related to clinician-directed diagnostic testing.

Documented conditions associated with secondary immune dysfunction were uncommon and were present in 10 patients overall, including 7 patients with localized pocket infection and 3 with systemic CIED infection. Six patients had documented current immunosuppressive or immunomodulatory treatment. Immunoglobulin concentrations were not routinely measured; therefore, hypogammaglobulinemia could be assessed only when explicitly documented or when testing was available. These variables were reported descriptively because of the small numbers in individual categories. Documented therapeutic antimicrobial exposure before the first blood culture or index biomarker assessment was identified in 13 patients. Among the 58 patients with blood cultures, the first culture was obtained before new therapeutic antimicrobial treatment in 40 patients, after treatment had started in 6 patients, and at an indeterminate time in 12 patients. Procalcitonin was available for 70 patients; the timing of measurement was before or at initiation of new therapeutic antimicrobial treatment in 49 patients, after treatment initiation in 8 patients, and not reliably reconstructable in 13 patients (Supplementary Table S6).

Analytical characteristics of the CRP and PCT assays, including assay platform, measuring interval, lower limit of detection, institutional reference range, and index sampling chronology relative to initiation of infection-directed antimicrobial treatment, are provided in Supplementary Table S7.

Device type, lead burden, previous CIED procedures, procedure-related hematoma, documented peri-procedural antimicrobial prophylaxis, and antithrombotic treatment are summarized descriptively in Supplementary Table S8. Because of the limited number of systemic-infection events and sparse distributions across several device- and procedure-related categories, these variables were not entered into multivariable regression models.

## 4. Discussion

In the current study, we compared the clinical, laboratory, microbiological, and outcome characteristics of localized pocket infection and systemic CIED infection. Systemic infection accounted for 18.7% of the study population. Fever and higher PCT concentrations were associated with the systemic-infection classification in exploratory models. Culture positivity and *Staphylococcus aureus* isolation were more frequent in systemic infection, while CoNS predominated in localized infections. Although device removal was performed in most patients with systemic infection, the median time to removal was longer in this group. Finally, all in-hospital deaths occurred among patients with systemic infection, emphasizing the prognostic importance of distinguishing systemic involvement from isolated pocket disease.

Fever was substantially more common in systemic than localized infection (35.7% vs. 4.9%) and remained independently associated with systemic infection in the Firth penalized model (aOR, 6.17; CI, 1.02–37.52). Nevertheless, nearly two-thirds of patients with systemic infection were afebrile, indicating that absence of fever cannot reliably exclude intravascular involvement. Conversely, localized findings such as drainage, erythema, tenderness, and edema were not sufficient to distinguish the two phenotypes. Pocket drainage was numerically more frequent in localized infection, but this difference was not statistically significant. Distinct clinical courses and prognoses of localized and systemic CIED infections have been described, with systemic infection carrying a markedly greater mortality risk [26]. This distinction supports our observation that systemic infection may occur without prominent pocket manifestations and that local examination alone is inadequate for determining infection extent. The clinical presentation of CIED infection can range from subtle pocket abnormalities to bacteremia, lead infection, and valvular endocarditis [2]. Multiple blood cultures and appropriate echocardiographic evaluation are recommended whenever systemic involvement is suspected, even when constitutional manifestations are limited [1].

TEE was not performed systematically in all patients and was undertaken more frequently in those ultimately classified as having systemic CIED infection. This pattern should be interpreted as reflecting clinician-directed diagnostic work-up rather than as an independent association with systemic disease. The decision to obtain blood cultures or perform TEE was likely influenced by fever, inflammatory-marker elevation, the perceived risk of bacteremia or endovascular involvement, clinical suspicion of lead or valve infection, and transthoracic echocardiographic findings. Consequently, patients who underwent a more extensive diagnostic evaluation had a greater opportunity to fulfil the microbiological or imaging components of the operational definition of systemic infection. Conversely, occult lead or valve involvement may have remained unrecognised in some patients classified as having localized infection who did not undergo TEE.

This differential diagnostic work-up introduces partial-verification bias, while the inclusion of positive blood cultures and echocardiographic vegetation in the systemic-infection definition introduces incorporation bias. Therefore, TEE use, vegetation detection, blood-culture positivity, and the composite measure of any positive microbiological culture should not be interpreted as independent discriminators or predictors of systemic infection in this cohort. These variables are best regarded as components of the diagnostic classification, and the observed associations of fever and PCT with systemic infection should be interpreted cautiously in this retrospective, clinician-directed diagnostic setting [1,2,8].

Among the investigated biomarkers, PCT showed the clearest group-level association with systemic infection. Median PCT was higher in systemic than in localized infection (0.23 vs. 0.05 ng/mL), whereas CRP and leukocyte counts were numerically higher but did not differ significantly between groups. In the kidney-function sensitivity model that included CKD stage G3a–G5, the procalcitonin point estimate remained directionally similar but its confidence interval included the null value (aOR per doubling 1.31, 95% CI 0.99–1.74; *p* = 0.058). This attenuation indicates that kidney dysfunction may partly confound the observed association between PCT and systemic infection; consequently, the PCT findings should be considered exploratory rather than evidence of an independent diagnostic effect. Thus, PCT may provide complementary information regarding the extent of an established CIED infection, whereas CRP and leukocyte count appear to have more limited value for differentiating systemic involvement in this cohort.

A contemporary extraction cohort including 88 patients with systemic infection and 68 with isolated pocket infection similarly found higher PCT concentrations in systemic infection, whereas PCT did not differ between isolated pocket infection and noninfected controls [27]. In that cohort, leukocyte-count elevation was limited to the comparison between systemic infection and controls, reinforcing the limited ability of routine inflammatory markers to exclude localized CIED infection. Localized biofilm-associated infection may therefore produce only a modest systemic inflammatory response despite clear pocket involvement.

PCT should be interpreted as an adjunctive marker of bacterial inflammatory burden rather than as a CIED infection-specific diagnostic test. The 2024 AHA scientific statement recommends considering PCT together with clinical findings, microbiological evaluation, and echocardiographic or other imaging findings when CIED infection is suspected [1]. In prospective pocket-infection studies, a PCT threshold of 0.05 ng/mL showed moderate diagnostic performance when compared with noninfected controls, with sensitivities of 60% and 68% and specificities of 82% and 77%, respectively [28,29]. Importantly, this threshold was developed to support identification of selected pocket-infection cohorts rather than to distinguish systemic from localized CIED infection. A value at or near this threshold should therefore not be used as a universal cut-off for systemic disease. Increasing PCT may strengthen clinical suspicion of bacteremia or endovascular involvement, but a low value should not be used to rule out localized or systemic CIED infection or to defer appropriate blood-culture and imaging assessment. Renal dysfunction, concomitant infection, recent procedures, and other sources of systemic inflammation should also be considered when interpreting an individual PCT result.

Microbiological cultures were positive in 44.0% of the overall cohort, with a significantly higher yield in systemic than localized infection (78.6% vs. 36.1%). Among culture-positive patients, CoNS was the most frequent pathogen, accounting for 42.4% of isolates, followed by methicillin-susceptible *S. aureus*. CoNS was predominantly associated with localized infection, whereas *S. aureus* represented more than half of culture-positive systemic infections. Within the prospective WRAP-IT population, staphylococci were the predominant pathogens, accounting for most microbiologically documented major CIED infections [30]. This microbiological pattern is consistent with the predominance of CoNS and *S. aureus* in our cohort. The ability of staphylococci to adhere to prosthetic material and form biofilm provides a biological explanation for their dominant role.

In a cohort of 219 patients, coagulase-negative staphylococci were identified in 45.2% of all CIED infections and in 68.3% of culture-positive infections [31]. Gram-negative bacilli were reported in 13.8% of culture-positive cases in the same cohort [31]. Our corresponding proportions—42.4% for CoNS and 12.1% for Gram-negative organisms—are broadly comparable, although CoNS was less dominant among our systemic infections. All four Gram-negative isolates in our study occurred in localized infection, but their small number prevents firm conclusions.

Systemic infections have also been reported to be more frequently monomicrobial, with *S. aureus* particularly associated with early systemic infection [26]. This agrees with the greater representation of *S. aureus* in our systemic group. The association is clinically plausible because *S. aureus* has greater invasive potential than most CoNS species and is more likely to produce bacteremia, lead infection, and endocarditis. Nevertheless, microbiological culture results should be interpreted cautiously because positive blood cultures contributed directly to the systemic infection definition. Furthermore, prior antibiotic treatment, biofilm formation, and differences in specimen quality may have contributed to culture-negative cases.

Rare pathogens identified in the systemic group—including *Enterococcus faecalis*, *Brucella* species, and *Candida albicans*—demonstrate the microbiological diversity of CIED infections in referred patient populations. These individual cases are clinically important because they may require organism-specific antimicrobial treatment and prolonged therapy. However, their frequency was too low to support pathogen-specific statistical comparisons.

Traditional comorbidities did not demonstrate robust associations with systemic rather than localized infection in our study. A meta-analysis has shown that diabetes, renal insufficiency, end-stage renal disease, heart failure, malignancy, corticosteroid exposure, and chronic obstructive pulmonary disease increase the overall risk of developing CIED infection [5]. These results do not necessarily conflict with ours because their meta-analysis examined infection occurrence among device recipients, whereas our analysis examined infection phenotype among patients who had already developed an infection. Risk factors for acquiring CIED infection may differ from those associated with progression from pocket infection to systemic disease. The limited number of systemic cases also reduced statistical power to identify moderate associations with comorbid conditions.

Device removal was documented in any system removal: 62/75 (82.7%); complete system removal: 58/75 (77.3%) of patients with systemic infection. However, the retrospective records did not permit reliable reconstruction of antimicrobial regimen, total treatment duration, clinical or microbiological response, or the case-specific indication for each removal procedure. Consequently, we cannot determine whether an individual procedure was undertaken because of persistent infection despite appropriate antimicrobial therapy, as primary source control after the diagnosis of definite CIED infection, or for another clinical reason. Importantly, device removal should not be interpreted as evidence that antimicrobial therapy was ineffective. In definite CIED infection, complete system removal is recommended as source control because antimicrobial therapy alone may not reliably eradicate infection involving implanted hardware and biofilm [1,2]. The longer interval to removal observed in the systemic group should therefore be interpreted as a descriptive management finding rather than evidence of delayed response to antimicrobial treatment.

Contemporary guidance, including the 2026 HRS/AHA/APHRS/EHRA/IDSA/LAHRS/PACES/STS expert consensus statement, supports complete removal of the device, all leads, and retained lead fragments for both definite localized and systemic CIED infection, ideally without unnecessary delay [2,32]. In combination with pathogen-directed antimicrobial therapy, early and complete hardware removal remains the cornerstone of treatment [1,32]. In a cohort of 416 patients, antibiotic treatment without device removal was associated with an approximately sevenfold increase in 30-day mortality; immediate removal was also associated with lower one-year mortality than delayed or absent removal [33]. A subsequent analysis of 11,304 Medicare patients with CIED infection found that extraction within six days was associated with lower mortality than no early extraction (adjusted hazard ratio, 0.69; 95% CI, 0.61–0.78) [34]. These studies make the longer removal interval observed in our systemic group an important target for quality improvement. However, our observational data cannot establish that the delay caused adverse outcomes, particularly because only two deaths occurred and sicker patients may inherently require longer stabilization and planning.

In-hospital mortality was 2.7% overall but 14.3% in systemic infection, whereas no deaths occurred in localized infection. Although the absolute number of deaths was small, the exclusive occurrence of mortality in systemic infection is consistent with the established prognostic gradient between localized pocket disease and endovascular infection. In a prior cohort, 30-day mortality rates were 8.9% for early systemic infection and 21.7% for delayed systemic infection, compared with no deaths among patients with early localized infection [26]. Endovascular infection was also independently associated with approximately twice the risk of one-year mortality compared with pocket infection, even after device removal [35]. This association supports the direction and magnitude of the difference observed in our cohort. The higher mortality of systemic infection probably reflects bacteremia, endocardial involvement, sepsis, greater comorbidity burden, and delays in achieving definitive source control rather than vegetation alone.

As clinical implications, Fever and elevated procalcitonin may help identify patients with CIED infection who require urgent evaluation for systemic involvement. However, normal inflammatory markers or absence of fever should not exclude systemic infection. Blood cultures and TEE should therefore be obtained promptly when clinically indicated, and confirmed infections should be managed with early complete device removal and pathogen-directed antimicrobial therapy.

Several limitations should be considered. First, this was a retrospective, single-center study with only 14 systemic infections and two in-hospital deaths. The Firth penalized method reduced small-sample bias but could not eliminate imprecision, as demonstrated by the wide confidence interval for fever. Second, the definitions of systemic infection incorporated positive blood cultures and echocardiographic vegetations. Associations involving culture positivity, TEE, and vegetation are therefore partly inherent to the classification system. Third, TEE was not performed uniformly, potentially leading to misclassification of occult systemic infection as localized disease; vegetation size, mobility, and embolic events were not systematically captured, precluding analyses based on a >20-mm threshold or recurrent embolism. Fourth, although documented immune-status conditions, immunosuppressive treatment, and prior antimicrobial exposure were re-abstracted where available, these data were not collected prospectively and could not be completely reconstructed for every patient. Immunoglobulin concentrations and formal nutritional assessments were not routinely available, and absence of documentation could not be interpreted as absence of immune dysfunction. Similarly, antimicrobial agent, route, treatment duration, and exact timing relative to cultures or biomarker measurement remained incomplete in a subset of patients. These limitations may have influenced microbiological yield and inflammatory-biomarker concentrations and preclude a definitive assessment of immune dysfunction or prior antimicrobial therapy as independent determinants of systemic infection. Fifth, PCT was unavailable in five patients, while ESR and HbA1c had substantially greater missingness. Cytokine measurements, including interleukin-6, were not available because stored biological samples were not retained for retrospective testing. In addition, absolute neutrophil and lymphocyte counts from the same index blood sample were not consistently available; therefore, NLR and SII could not be calculated reliably. Prospective studies using standardized pre-treatment sampling should evaluate whether cytokine and differential blood-count-based biomarkers provide incremental value beyond PCT and routine inflammatory markers. Sixth, detailed information on antimicrobial regimens, route, timing, total treatment duration, clinical or microbiological response, and standardized indications for device removal was not consistently available. We could therefore not assess adherence to syndrome-specific antimicrobial treatment recommendations or determine whether individual device removals followed persistent infection despite appropriate antimicrobial therapy. Information on extraction completeness, procedural complications, reimplantation strategy, and long-term recurrence was also limited. Finally, only in-hospital mortality was evaluated; delayed mortality and recurrent infection could not be assessed.

Despite these limitations, the study provides a clinically relevant comparison of two important CIED infection phenotypes and incorporates a rare-event regression approach appropriate for the limited number of systemic cases. Our findings suggest that fever and increasing PCT may help identify patients requiring urgent evaluation for systemic involvement. However, neither absence of fever nor low inflammatory markers should be used to exclude systemic infection. Prompt blood cultures, appropriate TEE, careful microbiological sampling, and early complete system removal remain essential. Larger prospective multicenter studies are required to validate the predictive value of fever and PCT and to determine whether biomarker-supported triage can shorten the time to extraction and improve outcomes.

## 5. Conclusions

In this retrospective single-center cohort, fever and higher procalcitonin concentrations were associated with the classification of systemic CIED infection. The absence of statistically detectable differences in CRP or leukocyte count should not be interpreted as evidence that these markers lack diagnostic value. The exploratory regression findings do not establish diagnostic discrimination, clinical prediction, or effects independent of kidney function, heart-failure severity, immune status, or other potential confounders. In-hospital mortality was observed only in the systemic-infection group, but this finding was based on two deaths and is strictly descriptive. Larger prospective cohorts with standardized diagnostic work-up, prespecified biomarker thresholds, and external validation are required.

## Figures and Tables

**Table 1:** Baseline and pre-classification characteristics according to final CIED infection classification.

Variable	Overall (n = 75)	Localized Pocket Infection (n = 61)	Systemic CIED Infection (n = 14)	*p*-Value
**Demographic and device-related characteristics**				
Age, years	66.8 ± 13.2	65.7 ± 13.4	71.6 ± 11.5	0.143
Male sex	52 (69.3)	41 (67.2)	11 (78.6)	0.529
Time since most recent CIED procedure, days	92 [24–333]	98 [26–347]	61 [17–203]	0.112
Pacemaker	42 (56.0)	35 (57.4)	7 (50.0)	0.890 ^a^
Implantable cardioverter-defibrillator	18 (24.0)	14 (23.0)	4 (28.6)	
Cardiac resynchronization therapy device	15 (20.0)	12 (19.7)	3 (21.4)	
**Comorbid conditions**				
Diabetes mellitus	43 (57.3)	33 (54.1)	10 (71.4)	0.370
Hypertension	57 (76.0)	44 (72.1)	13 (92.9)	0.165
Coronary artery disease	44 (58.7)	34 (55.7)	10 (71.4)	0.377
Heart failure	51 (68.0)	38 (62.3)	13 (92.9)	0.029 *
Chronic kidney disease, stage G3a–G5 ^b^	5 (6.7)	2 (3.3)	3 (21.4)	0.042 *
Estimated glomerular filtration rate, mL/min/1.73 m^2^	86 [69–98]	89 [73–99]	64 [45–83]	0.014 *
Dialysis-dependent kidney disease	1 (1.3)	0 (0.0)	1 (7.1)	0.187
**Pre-classification clinical and laboratory variables**				
Fever at presentation ^c^	8 (10.7)	3 (4.9)	5 (35.7)	0.005 **
Pocket drainage at presentation	40 (53.3)	35 (57.4)	5 (35.7)	0.234
Procalcitonin available	70 (93.3)	57 (93.4)	13 (92.9)	1.000
Procalcitonin, ng/mL	0.06 [0.03–0.15]	0.05 [0.03–0.12]	0.23 [0.06–1.57]	0.025 *

Data are presented as n (%), mean ± standard deviation, or median [interquartile range]. Categorical variables were compared using Fisher’s exact test, continuous variables using the independent-samples *t*-test or Mann–Whitney U test, as appropriate. * *p* < 0.05; ** *p* < 0.01. ^a^ Global *p*-value for device category. ^b^ Chronic kidney disease was defined as documented chronic kidney disease with estimated glomerular filtration rate < 60 mL/min/1.73 m^2^ for ≥3 months or dialysis dependence. ^c^ Temperature ≥ 38.0 °C at admission or initial infection assessment, before initiation of new infection-directed antimicrobial treatment when available.

**Table 2:** Clinical presentation, laboratory parameters, and imaging findings according to CIED infection type.

Variable	Overall Cohort (n = 75)	Localized CIED Infection (n = 61)	Systemic CIED Infection (n = 14)	*p*-Value
**Clinical presentation**				
Fever at presentation	8 (10.7)	3 (4.9)	5 (35.7)	0.005 **
Pocket drainage at presentation	40 (53.3)	35 (57.4)	5 (35.7)	0.234
Procalcitonin, ng/mL	0.06 [0.03–0.15]	0.05 [0.03–0.12]	0.23 [0.06–1.57]	0.025 *
Transthoracic echocardiography performed	73 (97.3)	59 (96.7)	14 (100.0)	1.000
Transesophageal echocardiography performed	25 (33.3)	13 (21.3)	12 (85.7)	<0.001 **
Fever at presentation	8 (10.7)	3 (4.9)	5 (35.7)	0.005 **
**Laboratory parameters**				
Leukocyte count, ×10^9^/L	8.03 [6.70–9.36]	7.94 [6.62–9.12]	8.75 [7.48–10.84]	0.164
Hemoglobin, g/dL	12.34 ± 2.13	12.33 ± 2.15	12.40 ± 2.11	0.908
Platelet count, ×10^9^/L	212 [159–260]	213 [164–300]	199 [112–238]	0.086
Erythrocyte sedimentation rate, mm/h ^a^	18.5 [11.0–37.8]	18.0 [11.0–37.8]	23.0 [15.8–44.0]	0.430
C-reactive protein, mg/L	7.1 [2.24–23.65]	6.62 [2.31–19.25]	24.70 [2.34–125.75]	0.209
Procalcitonin, ng/mL ^b^	0.05 [0.03–0.14]	0.05 [0.03–0.12]	0.23 [0.06–1.57]	0.022 *
HbA1c, % ^c^	8.1 [6.8–10.0]	8.1 [6.8–10.2]	8.5 [7.1–9.4]	0.742
**Imaging findings**				
Transthoracic echocardiography performed	80 (97.6)	66 (97.1)	14 (100.0)	1.000
Transesophageal echocardiography performed	25 (30.5)	13 (19.1)	12 (85.7)	<0.001
Echocardiographic vegetation	10 (12.2)	0 (0.0)	10 (71.4)	—
Pacemaker/ICD lead vegetation	5 (6.1)	0 (0.0)	5 (35.7)	—
Tricuspid-valve vegetation	4 (4.9)	0 (0.0)	4 (28.6)	—
Vena-cava vegetation	2 (2.4)	0 (0.0)	2 (14.3)	—
Aortic-valve vegetation	1 (1.2)	0 (0.0)	1 (7.1)	—

^a^ Erythrocyte sedimentation rate was available for 48 patients (40 localized and 8 systemic infections). ^b^ Procalcitonin was available for 70 patients (57 localized and 13 systemic infections). ^c^ HbA1c was available for 41 patients (33 localized and 8 systemic infections). Column headings report the total number of patients in each study group and do not indicate the number of laboratory tests. For laboratory parameters, each value represents the first available measurement obtained at admission or initial infection assessment for one patient and is summarized among patients with an available result. Leukocyte count, hemoglobin, platelet count, and C-reactive protein were available for all patients (overall, 75; localized, 61; systemic, 14).

**Table 3:** Descriptive microbiological findings, device management, and in-hospital outcomes according to final CIED infection classification.

Variable	Overall (n = 75)	Localized CIED Infection (n = 61)	Systemic CIED Infection (n = 14)	*p*-Value
**Microbiological** **sampling and culture results**				
Pocket-drainage culture obtained, n (%)	39 (52.0)	35 (57.4)	4 (28.6)	—
Positive pocket-drainage culture, n/N (%)	16/39 (41.0)	14/35 (40.0)	2/4 (50.0)	—
Intraoperative pocket tissue or extracted device-component culture obtained, n (%)	50 (66.7)	39 (63.9)	11 (78.6)	—
Positive intraoperative culture, n/N (%)	16/50 (32.0)	12/39 (30.8)	4/11 (36.4)	—
Blood culture obtained, n (%) ^a^	58 (77.3)	44 (72.1)	14 (100.0)	—
Clinically significant positive blood culture, n/N (%) ^a^	8/58 (13.8)	0/44 (0.0)	8/14 (57.1)	—
Any positive microbiological culture, n (%) ^a, b^	33 (44.0)	22 (36.1)	11 (78.6)	—
**Primary clinically relevant microorganism among culture-positive patients**				
*Staphylococcus aureus*, n/N (%)	12/33 (36.4)	6/22 (27.3)	6/11 (54.5)	—
Methicillin-susceptible *S. aureus*, n/N (%)	11/33 (33.3)	6/22 (27.3)	5/11 (45.5)	—
Methicillin-resistant *S. aureus*, n/N (%)	1/33 (3.0)	0/22 (0.0)	1/11 (9.1)	—
Coagulase-negative staphylococci, n/N (%)	14/33 (42.4)	12/22 (54.5)	2/11 (18.2)	—
Gram-negative bacteria, n/N (%) ^c^	4/33 (12.1)	4/22 (18.2)	0/11 (0.0)	—
Other microorganisms, n/N (%) ^d^	3/33 (9.1)	0/22 (0.0)	3/11 (27.3)	—
**Device management during index hospitalization**				
Complete system removal	58 (77.3)	46 (75.4)	12 (85.7)	—
Incomplete/partial system removal with retained hardware	4 (5.3)	3 (4.9)	1 (7.1)	—
Any system removal	62 (82.7)	49 (80.3)	13 (92.9)	—
Pocket debridement without system removal	3 (4.0)	3 (4.9)	0 (0.0)	—
Conservative treatment without attempted system removal	10 (13.3)	9 (14.8)	1 (7.1)	—
Time from index admission to first removal attempt, days *	6 [3–9]	5 [3–8]	10 [6–17]	0.026 *
**In-hospital outcomes**				
In-hospital mortality, n (%)	2 (2.7)	0 (0.0)	2 (14.3)	—

Management categories are mutually exclusive. Complete system removal indicates removal of the pulse generator and all transvenous leads without a documented retained lead fragment. Incomplete/partial system removal indicates a removal attempt with retained lead material or another device component. * Calculated among the 62 patients who underwent any system removal; time was measured from index hospital admission to the first removal attempt. Device-management categories are descriptive because treatment selection was not randomized and detailed antimicrobial-response data were not consistently available. * *p* < 0.05. ^a^ For specimen-specific culture positivity, percentages were calculated among patients from whom the corresponding specimen was obtained. Blood-culture positivity contributed to the classification of systemic CIED infection; therefore, microbiological findings are presented descriptively without between-group inferential comparisons. ^b^ For microorganism distributions, percentages were calculated among culture-positive patients: 33 overall, 22 with localized pocket infection, and 11 with systemic CIED infection. Methicillin-susceptible and methicillin-resistant *Staphylococcus aureus* are subcategories of the total *S. aureus* count. ^c^ Gram-negative bacteria comprised *Pseudomonas* spp., *Achromobacter xylosoxidans*, *Moraxella osloensis*, and *Enterobacter aerogenes*, with one case each. ^d^ Other microorganisms comprised *Enterococcus faecalis*, *Brucella* spp., and *Candida albicans*, with one systemic-infection case each.

**Table 4:** Firth penalized logistic regression analyses of pre-classification factors associated with systemic CIED infection.

**Panel A. Univariable Firth Penalized Logistic Regression**				
**Variable**		**Analytic Sample,** **n (Systemic Events)**	**OR (95% CI)**	***p*** **-Value**
Age, per 10-year increase		75 (14)	1.40 (0.86–2.29)	0.172
Male sex		75 (14)	1.66 (0.46–5.97)	0.440
Diabetes mellitus		75 (14)	1.94 (0.55–6.80)	0.300
Heart failure		75 (14)	6.45 (0.77–53.90)	0.086
Chronic kidney disease, stage G3a–G5		75 (14)	7.12 (1.09–46.40)	0.042 *
Time since most recent CIED procedure, per doubling		75 (14)	0.81 (0.63–1.05)	0.112
Pocket drainage at presentation		75 (14)	0.44 (0.13–1.50)	0.193
Fever at presentation		75 (14)	10.74 (2.17–53.20)	0.003 **
Procalcitonin, per doubling		70 (13)	1.46 (1.11–1.94)	0.008 **
**Panel** **B. Exploratory Multivariable Firth Penalized Logistic Regression**				
**Variable**	**Model A: Age, Fever, and** **log_2_ (PCT) (n = 70; 13 Events)** **aOR (95% CI)**	***p*** **-** **Value**	**Model B: CKD, Fever, and** **log_2_ (PCT) (n = 70; 13 Events)** **aOR (95% CI)**	***p*** **-Value**
Age, per 10-year increase	1.23 (0.72–2.11)	0.450	—	—
Chronic kidney disease, stage G3a–G5	—	—	4.15 (0.39–44.00)	0.240
Fever at presentation	6.17 (1.02–37.52)	0.048 *	5.82 (0.91–37.00)	0.064
Procalcitonin, per doubling	1.35 (1.02–1.81)	0.039 *	1.31 (0.99–1.74)	0.058

Systemic CIED infection was the dependent outcome (coded 1); localized pocket infection was the reference category (coded 0). All estimates were obtained using Firth penalized logistic regression. Procalcitonin and time since the most recent CIED procedure were log_2_-transformed; their odds ratios therefore represent the change in odds associated with a twofold increase. Models including procalcitonin used complete-case analysis. Model B was a prespecified exploratory sensitivity model to assess potential confounding by kidney dysfunction. Blood-culture positivity, echocardiographic vegetation, TEE use, microbiological isolate, device removal, time to removal, and mortality were not entered because they contributed to diagnostic classification or occurred after the index clinical presentation. * *p* < 0.05; ** *p* < 0.01.

## Data Availability

The original contributions presented in this study are included in the article/Supplementary Material. Further inquiries can be directed to the corresponding author.

## Supplementary Materials

The following supporting information can be downloaded at: https://www.mdpi.com/article/10.3390/diagnostics16183062/s1, Table S1: Diagnostic re-adjudication of patients without device removal; Table S2: Heart-failure phenotype, severity, CRT indication, and kidney-disease stratification according to final CIED infection classification; Table S3: Availability and missingness of clinical, laboratory, imaging, and microbiological variables included in the final analytic cohort; Table S4: Distribution of available admission laboratory measurements relative to institutional reference intervals according to CIED infection type; Table S5: Diagnostic work-up, bloodstream-infection adjudication, and strict-verification sensitivity analysis according to final CIED infection classification; Table S6: Documented immune-status conditions, immunosuppressive treatment, and antimicrobial exposure according to final CIED infection classification; Table S7: Analytical characteristics and index sampling chronology of C-reactive protein and procalcitonin; Table S8: Device- and procedure-related characteristics according to final CIED infection classification.

## Abbreviations

aOR	Adjusted odds ratio
CI	Confidence interval
CIED	Cardiac implantable electronic device
CoNS	Coagulase-negative staphylococci
CRP	C-reactive protein
CRT	Cardiac resynchronization therapy
ESR	Erythrocyte sedimentation rate
HbA1c	Glycated hemoglobin
ICD	Implantable cardioverter-defibrillator
WBC	White blood cell count
IQR	Interquartile range
MRSA	Methicillin-resistant *Staphylococcus aureus*
MSSA	Methicillin-susceptible *Staphylococcus aureus*
OR	Odds ratio
PCT	Procalcitonin
SPSS	Statistical Package for the Social Sciences
TEE	Transesophageal echocardiography
TTE	Transthoracic echocardiography
