# Supplementary material for: Beyond the Pocket: Clinical, Routine Biomarker, and Microbiological Associations with Systemic Versus Localized Cardiovascular Implantable Electronic Device Infection
Source: Diagnostics (Basel). 2026 Sep 21;16(18):3062. doi: 10.3390/diagnostics16183062 (PMC13605586; doi:10.3390/diagnostics16183062)
Supplement: Supplementary file 1 [file diagnostics-16-03062-s001.zip › diagnostics-4511757-supplementary.pdf]

Supplementary Table S1. Diagnostic re-adjudication of patients without device removal.

Initial classification in the 82-patient cohort	Final classification after re-adjudication	n (% of 20)	Basis for classification	Included in final primary cohort
Localized pocket infection (n = 19)	Definite localized pocket CIED infection	12 (60.0)	Documented generator-pocket or hardware involvement without systemic features	Yes
Localized pocket infection (n = 19)	Superficial incisional infection	5 (25.0)	Acute inflammation confined to skin/subcutaneous tissue; no documented pocket or hardware involvement	No
Localized pocket infection (n = 19)	Possible or indeterminate infection	2 (10.0)	Available records did not permit reliable differentiation between superficial inflammation and definite CIED infection	No
Systemic CIED infection (n = 1)	Definite systemic CIED infection	1 (5.0)	Device-attributable bloodstream infection and/or compatible lead or valvular involvement	Yes
**Total**		20 (100.0)		13 retained; 7 excluded

Diagnostic re-adjudication used prespecified criteria derived from the EHRA International CIED Infection Criteria and the 2023 ESC Guidelines. Device-removal status and time from implantation were not used as diagnostic criteria. Of the 20 patients without documented device removal in the preliminary cohort, 13 fulfilled criteria for definite CIED infection and were retained in the final cohort; 7 were excluded after re-adjudication.

Supplementary Table S2. Heart-failure phenotype, severity, CRT indication, and kidney-disease stratification according to final CIED infection classification.

Variable	Overall	Localized pocket infection	Systemic CIED infection
**Heart-failure assessment**			
Heart failure	51/75 (68.0)	38/61 (62.3)	13/14 (92.9)
HFrEF	19/51 (37.3)	14/38 (36.8)	5/13 (38.5)
HFmrEF	11/51 (21.6)	8/38 (21.1)	3/13 (23.1)
HFpEF	21/51 (41.2)	16/38 (42.1)	5/13 (38.5)
LVEF available	48/51 (94.1)	35/38 (92.1)	13/13 (100.0)
LVEF, %	46 [34–55]	48 [36–56]	40 [30–48]
NYHA class available	45/51 (88.2)	33/38 (86.8)	12/13 (92.3)
NYHA class III–IV	16/45 (35.6)	10/33 (30.3)	6/12 (50.0)
Acute decompensated heart failure at index presentation	7/51 (13.7)	4/38 (10.5)	3/13 (23.1)
CRT device	15/75 (20.0)	12/61 (19.7)	3/14 (21.4)
CRT implanted for chronic HF/dyssynchrony indication	13/15 (86.7)	10/12 (83.3)	3/3 (100.0)
CRT implanted because of anticipated high right-ventricular pacing burden or other indication	2/15 (13.3)	2/12 (16.7)	0/3 (0.0)
**Kidney-function assessment**			
eGFR available at index assessment	75/75 (100.0)	61/61 (100.0)	14/14 (100.0)
eGFR, mL/min/1.73 m^2	86 [69–98]	89 [73–99]	64 [45–83]
CKD G3a	3/75 (4.0)	2/61 (3.3)	1/14 (7.1)
CKD G3b	1/75 (1.3)	0/61 (0.0)	1/14 (7.1)
CKD G4	0/75 (0.0)	0/61 (0.0)	0/14 (0.0)
CKD G5/dialysis-dependent kidney disease	1/75 (1.3)	0/61 (0.0)	1/14 (7.1)
Any CKD G3a–G5	5/75 (6.7)	2/61 (3.3)	3/14 (21.4)
Advanced CKD (G4–G5) or maintenance dialysis	1/75 (1.3)	0/61 (0.0)	1/14 (7.1)

Data are presented as n/N (%) or median [interquartile range]. Heart-failure phenotype was assigned using the most recent clinically stable LVEF. HFrEF, HF with reduced ejection fraction; HFmrEF, HF with mildly reduced ejection fraction; HFpEF, HF with preserved ejection fraction; CRT, cardiac resynchronization therapy; eGFR, estimated glomerular filtration rate; CKD, chronic kidney disease; NYHA, New York Heart Association. Because of sparse subgroup counts, these descriptive data should not be interpreted as independent between-group associations.

Supplementary Table S3. Availability and missingness of clinical, laboratory, imaging, and microbiological variables included in the study.

Domain	Variable	Available, n (%)	Missing, n (%)
**Classification Demographic and clinical variables**	CIED infection type	82 (100.0)	0 (0.0)
	Age	82 (100.0)	0 (0.0)
	Sex	82 (100.0)	0 (0.0)
	Time since most recent device procedure	82 (100.0)	0 (0.0)
	Fever	82 (100.0)	0 (0.0)
	Pocket drainage	82 (100.0)	0 (0.0)
	Pocket edema	82 (100.0)	0 (0.0)
	Pocket tenderness	82 (100.0)	0 (0.0)
	Pocket erythema	82 (100.0)	0 (0.0)
**Comorbidities**	Diabetes mellitus	82 (100.0)	0 (0.0)
	Hypertension	82 (100.0)	0 (0.0)
	Chronic kidney disease	82 (100.0)	0 (0.0)
	Chronic obstructive pulmonary disease	82 (100.0)	0 (0.0)
	Human immunodeficiency virus infection	82 (100.0)	0 (0.0)
	Coronary artery disease	82 (100.0)	0 (0.0)
	Heart failure	81 (98.8)	1 (1.2)
**Laboratory variables**	Leukocyte count	82 (100.0)	0 (0.0)
	Hemoglobin	82 (100.0)	0 (0.0)
	Platelet count	82 (100.0)	0 (0.0)
	Erythrocyte sedimentation rate	48 (58.5)	34 (41.5)
	C-reactive protein	82 (100.0)	0 (0.0)
	Procalcitonin	77 (93.9)	5 (6.1)
	HbA1c	41 (50.0)	41 (50.0)
**Imaging variables**	TTE performance status	81 (98.8)	1 (1.2)
	TTE findings among patients undergoing TTE	78 (97.5)	2 (2.5)
	TEE performance status	81 (98.8)	1 (1.2)
	TEE findings among patients undergoing TEE	25 (100.0)	0 (0.0)
	Echocardiographic vegetation status*	82 (100.0)	0 (0.0)
**Microbiological variables**	Pocket-drainage culture collection status	81 (98.8)	1 (1.2)
	Pocket-drainage culture result among sampled patients	44 (100.0)	0 (0.0)
	Intraoperative culture collection status	82 (100.0)	0 (0.0)
	Intraoperative culture result among sampled patients	53 (98.1)	1 (1.9)
	Blood-culture collection status	82 (100.0)	0 (0.0)
	Blood-culture result among sampled patients	65 (100.0)	0 (0.0)
	Microorganism identification among culture-positive patients	36 (100.0)	0 (0.0)
**Device management and outcome**	Device-removal status	82 (100.0)	0 (0.0)
	Time to device removal among patients undergoing removal	62 (100.0)	0 (0.0)
	In-hospital mortality	82 (100.0)	0 (0.0)

Availability percentages were calculated using the number of eligible observations for each variable. Conditional variables, such as imaging findings, culture results, microorganism identification, and time to device removal, were evaluated only among patients for whom the corresponding procedure or event occurred. Three nonnumeric erythrocyte sedimentation rate entries were considered missing. Echocardiographic vegetation status was derived from the combined TTE and TEE records.

Supplementary Table S4. Distribution of available admission laboratory measurements relative to reference intervals, according to CIED infection type.

Parameter	Institutional reference interval/definition	Category	Overall, n/N (%)	Localized pocket infection, n/N (%)	Systemic CIED infection, n/N (%)
Leukocyte count, $\times 10^9$/L	4.0–10.0	Below range	1/75 (1.3)	1/61 (1.6)	0/14 (0.0)
		Within range	59/75 (78.7)	50/61 (82.0)	9/14 (64.3)
		Above range	15/75 (20.0)	10/61 (16.4)	5/14 (35.7)
Hemoglobin, g/dL	Female: 12.0–16.0; male: 13.0–17.0	Below range	25/75 (33.3)	19/61 (31.1)	6/14 (42.9)
		Within range	49/75 (65.3)	41/61 (67.2)	8/14 (57.1)
		Above range	1/75 (1.3)	1/61 (1.6)	0/14 (0.0)
Platelet count, $\times 10^9$/L	150–400	Below range	11/75 (14.7)	8/61 (13.1)	3/14 (21.4)
		Within range	58/75 (77.3)	48/61 (78.7)	10/14 (71.4)
		Above range	6/75 (8.0)	5/61 (8.2)	1/14 (7.1)
Erythrocyte sedimentation rate, mm/h	Age- and sex-specific institutional upper limit	Within range	20/44 (45.5)	18/36 (50.0)	2/8 (25.0)
		Above range	24/44 (54.5)	18/36 (50.0)	6/8 (75.0)
C-reactive protein, mg/L	<5.0	Within range	29/75 (38.7)	27/61 (44.3)	2/14 (14.3)
		Above upper limit of normal	46/75 (61.3)	34/61 (55.7)	12/14 (85.7)
Procalcitonin, ng/mL	<0.05	Within range	38/70 (54.3)	36/57 (63.2)	2/13 (15.4)
		Above upper limit of normal	32/70 (45.7)	21/57 (36.8)	11/13 (84.6)
HbA1c, %	4.0–5.6	Within range	9/37 (24.3)	8/29 (27.6)	1/8 (12.5)
		Above upper limit of normal	28/37 (75.7)	21/29 (72.4)	7/8 (87.5)

Denominators represent patients with an available index measurement in the final re-adjudicated cohort. Laboratory results were the first available values obtained at admission or initial infection assessment, before or at initiation of infection-directed treatment where available. Reference intervals were those in use at the institutional laboratory during the study period. This descriptive table was not used to infer diagnostic accuracy or to define clinical biomarker cut-off values.

Supplementary Table S5. Diagnostic work-up, bloodstream-infection adjudication, and strict-verification sensitivity analysis according to final CIED infection classification.

Variable	Overall (n = 75)	Localized pocket infection (n = 61)	Systemic CIED infection (n = 14)
**A. Blood-culture assessment**			
At least one blood-culture set obtained, n (%)	58 (77.3)	44 (72.1)	14 (100.0)
One set obtained, n	9	9	0
Two sets obtained, n	20	18	2
At least three sets obtained, n	29	17	12
First culture obtained before new antimicrobial treatment, n/N (%)	40/58 (69.0)	28/44 (63.6)	12/14 (85.7)
First culture obtained after antimicrobial treatment had started, n/N (%)	6/58 (10.3)	5/44 (11.4)	1/14 (7.1)
Timing of first culture not reliably documented, n/N (%)	12/58 (20.7)	11/44 (25.0)	1/14 (7.1)
Documented antimicrobial exposure before first culture, n (%)	13 (17.3)	11 (18.0)	2 (14.3)
**B. Echocardiographic and additional imaging assessment**			
TTE performed, n (%)	73 (97.3)	59 (96.7)	14 (100.0)
TEE performed, n (%)	25 (33.3)	13 (21.3)	12 (85.7)
Time from presentation to TEE, days, median [IQR]	2 [1–3]	2 [1–3]	1 [0–2]
Repeat TTE and/or TEE, n (%)	7 (9.3)	3 (4.9)	4 (28.6)
Additional imaging, n (%)	3 (4.0)	2 (3.3)	1 (7.1)
**Documented indication for TEE***			
Bacteremia or positive blood cultures, n	12	2	10
Suspected lead or valvular involvement, n	11	4	7
Inconclusive or non-diagnostic TTE, n	5	4	1
Persistent fever or systemic inflammatory features, n	3	2	1
Pre-extraction procedural assessment, n	6	3	3
**C. Adjudication of bloodstream infection**			
Clinically significant positive blood culture, n (%)	8 (10.7)	0 (0.0)	8 (57.1)
Alternative non-CIED source actively assessed, n/N (%)	8/8 (100.0)	—	8/8 (100.0)
Plausible alternative source identified, n/N (%)	0/8 (0.0)	—	0/8 (0.0)
Device-attributable bloodstream infection after re-adjudication, n (%)	8 (10.7)	0 (0.0)	8 (57.1)
Systemic infection without positive blood culture, n (%)	6 (8.0)	0 (0.0)	6 (42.9)
**D. Strict-verification sensitivity cohort**			
Complete systemic-infection evaluation**, n (%)	20 (26.7)	8 (13.1)	12 (85.7)
Incompletely verified or not assessed, n (%)	55 (73.3)	53 (86.9)	2 (14.3)
Fever, n (%)	1 (12.5)	1 (12.5)	5 (41.7)
Procalcitonin, ng/mL, median [IQR]	0.16 [0.05–0.46]	0.07 [0.04–0.13]	0.28 [0.07–1.45]
Multivariable model	Not performed	—	—

Data are presented as n (%), n/N (%), or median [interquartile range]. *More than one indication could be recorded for the same patient; percentages are therefore not shown and row totals may exceed the number of patients undergoing TEE. **For this strict-verification analysis, complete systemic-infection evaluation required at least three separate peripheral blood-culture sets obtained before the first new therapeutic antimicrobial dose and completion of both TTE and TEE during the index diagnostic episode. This is a conservative research definition for sensitivity analysis, not a universal standard-of-care requirement. Positive blood-culture results and echocardiographic vegetation contributed to the operational classification of systemic CIED infection. Therefore, blood-culture positivity, TEE use, and the composite variable of any positive microbiological culture are reported descriptively and should not be interpreted as independent between-group associations. Tests not performed, or not reliably reconstructable, were coded as not assessed rather than negative.

Supplementary Table S6. Documented immune-status conditions, immunosuppressive treatment, and antimicrobial exposure according to final CIED infection classification.

**Variable**	**Overall (n = 75)**	**Localized pocket infection (n = 61)**	**Systemic CIED infection (n = 14)**
**Documented immune-status conditions**			
Any documented condition associated with secondary immune dysfunction	10 (13.3)	7 (11.5)	3 (21.4)
Primary immunodeficiency	0 (0.0)	0 (0.0)	0 (0.0)
Active or recent solid-organ malignancy	3 (4.0)	2 (3.3)	1 (7.1)
Hematological malignancy or bone-marrow disorder	1 (1.3)	0 (0.0)	1 (7.1)
Solid-organ transplantation	1 (1.3)	1 (1.6)	0 (0.0)
Documented hypogammaglobulinemia	0 (0.0)	0 (0.0)	0 (0.0)
Immunoglobulin concentration measured	3 (4.0)	3 (4.9)	0 (0.0)
Chronic liver disease or cirrhosis	3 (4.0)	3 (4.9)	0 (0.0)
Clinician-documented malnutrition or cachexia	2 (2.7)	1 (1.6)	1 (7.1)
**Immunosuppressive or immunomodulatory treatment**			
Any documented current treatment	6 (8.0)	4 (6.6)	2 (14.3)
Systemic corticosteroid exposure	3 (4.0)	2 (3.3)	1 (7.1)
Cytotoxic chemotherapy	1 (1.3)	0 (0.0)	1 (7.1)
Biologic or targeted immune-modifying treatment	1 (1.3)	1 (1.6)	0 (0.0)
Calcineurin inhibitor	1 (1.3)	1 (1.6)	0 (0.0)
**Antimicrobial exposure and sampling chronology**			
Documented therapeutic antimicrobial exposure before first blood culture or biomarker assessment	13 (17.3)	11 (18.0)	2 (14.3)
Blood cultures obtained	58 (77.3)	44 (72.1)	14 (100.0)
First blood culture before new therapeutic antimicrobial treatment*	40/58 (69.0)	28/44 (63.6)	12/14 (85.7)
First blood culture after antimicrobial treatment had started*	6/58 (10.3)	5/44 (11.4)	1/14 (7.1)
Timing of first blood culture not reliably reconstructable*	12/58 (20.7)	11/44 (25.0)	1/14 (7.1)
Procalcitonin available	70 (93.3)	57 (93.4)	13 (92.9)
Procalcitonin obtained before or at initiation of new therapeutic antimicrobial treatment**	49/70 (70.0)	40/57 (70.2)	9/13 (69.2)
Procalcitonin obtained after therapeutic antimicrobial treatment had started**	8/70 (11.4)	6/57 (10.5)	2/13 (15.4)
Procalcitonin-timing information not reliably reconstructable**	13/70 (18.6)	11/57 (19.3)	2/13 (15.4)

Data are presented as n (%) or n/N (%). Conditions and treatment exposures were recorded only when explicitly documented; absent documentation was not interpreted as absence. *Denominator: patients from whom at least one blood-culture set was obtained. **Denominator: patients with an available procalcitonin value. More than one immune-status condition or medication exposure could be present in an individual patient; subgroup totals should not be summed.

Supplementary Table S7. Analytical characteristics and index sampling chronology of C-reactive protein and procalcitonin.

Characteristic	C-reactive protein	Procalcitonin
Specimen	Serum	Serum
Assay manufacturer and method	Roche Diagnostics, Tina-quant CRP Gen. 3, immunoturbidimetric assay	Roche Diagnostics, Elecsys BRAHMS PCT, electrochemiluminescence immunoassay
Analyzer platform	cobas c 501 analyzer	cobas e 801 analyzer
Analytical measuring interval	0.3–350 mg/L	0.02–100 ng/mL
Lower limit of detection	0.3 mg/L	0.02 ng/mL
Institutional reference interval	<5.0 mg/L	<0.05 ng/mL
Index measurement	First available value obtained within 24 h of presentation or initial infection assessment	First available value obtained within 24 h of presentation or initial infection assessment
Relation to new infection-directed antimicrobial treatment	Sample obtained before or at treatment initiation whenever available; otherwise classified as post-treatment or timing unknown	Sample obtained before or at treatment initiation whenever available; otherwise classified as post-treatment or timing unknown
Availability in final analytic cohort	75/75 (100.0)	70/75 (93.3)
Sampling before or at new antimicrobial-treatment initiation	52/75 (69.3)	49/70 (70.0)
Sampling after treatment initiation	9/75 (12.0)	8/70 (11.4)
Timing not reliably reconstructable	14/75 (18.7)	13/70 (18.6)

Supplementary Table S8. Device- and procedure-related characteristics according to final CIED infection classification.

Variable	Overall (n = 75)	Localized pocket infection (n = 61)	Systemic CIED infection (n = 14)
**Device characteristics**			
Permanent pacemaker	42 (56.0)	35 (57.4)	7 (50.0)
Implantable cardioverter-defibrillator	18 (24.0)	14 (23.0)	4 (28.6)
CRT-P	6 (8.0)	5 (8.2)	1 (7.1)
CRT-D	9 (12.0)	7 (11.5)	2 (14.3)
Number of transvenous leads	2 [2–2]	2 [2–2]	2 [2–3]
Three or more leads	12 (16.0)	8 (13.1)	4 (28.6)
**Previous device procedures**			
Number of previous CIED procedures	1 [0–2]	1 [0–2]	1 [0–2]
Generator replacement before index infection	20 (26.7)	16 (26.2)	4 (28.6)
Lead revision before index infection	8 (10.7)	6 (9.8)	2 (14.3)
System upgrade before index infection	7 (9.3)	5 (8.2)	2 (14.3)
Temporary pacing during the most recent procedure	5 (6.7)	4 (6.6)	1 (7.1)
Procedure-related pocket hematoma	5 (6.7)	4 (6.6)	1 (7.1)
**Periprocedural treatment**			
Documented peri-procedural antimicrobial prophylaxis	69 (92.0)	56 (91.8)	13 (92.9)
Oral anticoagulant treatment at index presentation	22 (29.3)	17 (27.9)	5 (35.7)
Antiplatelet treatment at index presentation	29 (38.7)	23 (37.7)	6 (42.9)
Dual antiplatelet therapy at index presentation	6 (8.0)	5 (8.2)	1 (7.1)

Data are presented as n (%) or median [interquartile range]. Procedure-related variables were obtained from available operative reports and medical records. Absence of documentation was coded as not reliably assessable rather than absence of the exposure. CRT-P, cardiac resynchronization therapy pacemaker; CRT-D, cardiac resynchronization therapy defibrillator.
